# Sodium-hyaluronate mouthwash on radiotherapy-induced xerostomia: a randomised clinical trial

**DOI:** 10.1007/s00520-023-08090-x

**Published:** 2023-10-18

**Authors:** Cosimo Rupe, Alessia Basco, Gioele Gioco, Romeo Patini, Alberta Lucchese, Francesco Micciché, Mariangela Massaccesi, Carlo Lajolo

**Affiliations:** 1 Head and Neck Department, School of Dentistry, Fondazione Policlinico Universitario A. Gemelli—IRCCS, Università Cattolica del Sacro Cuore, Largo A. Gemelli, 8, 00168 Rome, Italy; 2 Multidisciplinary Department of Medical-Surgical and Dental Specialties, University of Campania “Luigi Vanvitelli”, Via Luigi de Crecchio 6, 80138 Naples, Italy; 3 Department of Radiation Oncology, Fondazione Policlinico Universitario A. Gemelli—IRCCS, Institute of Radiology, Università Cattolica del Sacro Cuore, Largo A. Gemelli, 8, 00168 Rome, Italy

**Keywords:** Radiotherapy, Xerostomia, Radiotherapy-induced xerostomia, GUM Hydral®, Mouthwash, Placebo

## Abstract

**Introduction:**

Radiotherapy-induced xerostomia (RIX) is one of the most common adverse effects of radiotherapy (RT) in head and neck cancer patients (HNC) and a major determinant of survivors’ quality of life. The primary objective was to evaluate the reduction of patients’ xerostomia symptoms after the utilisation of a sodium-hyaluronate mouthwash compared to a placebo solution. The secondary objectives were to evaluate the improvement of quality of life and to evaluate the patients’ satisfaction.

**Methods:**

The protocol was approved by the ethical committee (Ref. 50,053/19) and registered at ClinicalTrials.gov (ID: NCT05103124). The study was a double-blind randomised clinical trial (RCT) with a crossover design and was conducted at the Fondazione Policlinico Universitario A. Gemelli, Rome.

**Results:**

Thirty-two patients completed the study protocol. Lower values of the modified Xerostomia Questionnaire (XQ) were retrieved when comparing the baseline scores to the ones after the treatment, when compared with placebo (Mann–Whitney *U* test = 0.01); higher values of patients’ satisfaction (Likert scale) and modified XQ were retrieved for the sodium-hyaluronate mouthwash (Mann–Whitney *U* test = 0.001).

**Conclusions:**

This RCT highlights the advantages of treating RIX with the sodium-hyaluronate mouthwash since it seems to be clinically effective in reducing its symptoms, without any reported adverse events.

ClinicalTrials.gov: NCT05103124 in 17/10/2021.

**Supplementary Information:**

The online version contains supplementary material available at 10.1007/s00520-023-08090-x.

## Introduction

Head and neck cancers (HNC) have a reported incidence of more than 900,000 new cases per year [1]. The multidisciplinary clinical management of HNCs is challenging, as evidenced by the introduction of the Tumour Boards, a team of specialists, including head and neck surgeons, radiation oncologists, medical oncologists, nuclear physicians, and oncologist dentists [2, 3]. During the last few decades, radiotherapy (RT) has gained a crucial therapeutic role, being recommended for 60% of HNC patients, either for treatment alone or as an adjuvant [4]. Several adverse events may occur during (acute adverse events: mucositis and dermatitis) or after (late adverse events: hyposalivation, dental caries, dysgeusia, osteoradionecrosis, and trismus) RT [5–8].

RT-induced xerostomia (RIX) is one of the most common adverse effects of RT in patients with HNC and a major determinant of survivors’ quality of life (QoL) [9]. Among the patients undergoing RT, 63–93% experience irreversible salivary gland damage when the salivary glands are within the irradiated field [10]. Typical manifestations of these damages are reduced salivary secretion, which in turn can translate into a subjective sensation of dry mouth (xerostomia); oral discomfort; altered taste; difficulty in speaking, swallowing, and chewing; and an increased risk of dental disease [11, 12]. Although hyposalivation and xerostomia are well-known clinical conditions that cause a substantial reduction in QoL [13], there is currently poor evidence regarding the treatment of hyposalivation and xerostomia in patients with HNC [10]. Salivary substitutes are widely used to ameliorate oral dehydration [14]. Among these, sodium-hyaluronate mouthwash is a product based on hyaluronic acid and sodium citrate. It helps in rehydrating and protecting the oral tissues by covering them with a film, thus reducing xerostomia symptoms.

The above-mentioned agent has already been tested in retrospective and prospective clinical trials [15]; nevertheless, no randomised controlled clinical trial (RCT) including only HNC patients has been performed.

The primary objective of this RCT was to evaluate the reduction of patients’ xerostomia symptoms due to RT through a modified version of the Xerostomia Questionnaire (XQ) [13] after sodium-hyaluronate mouthwash administration when compared with placebo administration.

The secondary objectives of this study were (1) to evaluate the improvement in QoL, evaluated through the European Organisation for Research and Treatment of Cancer (EORTC) QLQ-C30 and QLQ-H&N35 questionnaires [16], and (2) to evaluate the patients’ satisfaction with the product through a Likert scale.

## Materials and methods

The protocol was approved by the ethics committee of the ‘Università Cattolica del Sacro Cuore’ (Ref. 50,053/19) and is registered at ClinicalTrials.gov (ID: NCT05103124). This study has been reported according to the CONSORT guidelines (Supplementary files, S1) [17].

### Trial design

The present study was conducted as a double-blind RCT with a crossover design; thus, the whole population received both the sodium-hyaluronate mouthwash and placebo.

### Participants

Patients with HNC who visited the Oral Medicine, Head and Neck Department – Fondazione Policlinico Universitario A. Gemelli—IRCSS between October 2020 and March 2022 were screened for inclusion. Patients were assessed during their routine follow-up within the context of the multidisciplinary Tumour Board.

The following inclusion criteria were applied:

- Patients older than 18 years.
- Patients diagnosed with HNC who had received local RT involving the salivary glands at least 3 months before the beginning of the study both for curative and palliative purposes, with or without chemotherapy, reporting xerostomia symptoms.
- Patients diagnosed with HNC who had received local RT as an adjuvant to surgical resection at least 3 months before the beginning of the study, with or without chemotherapy, reporting xerostomia symptoms.

The exclusion criteria were as follows:

- Patients with documented contraindications to any of the components of sodium-hyaluronate mouthwash (including excipients).
- Patients with neurological and psychiatric conditions influencing their ability to self-apply the treatment.
- Patients unwilling to complete the request diary card.
- Patients unable to attend the ambulatory visits scheduled by the protocol.
- Patients participating in other clinical studies.
- Patients who had received antitumour treatment during the previous 3 months.
- Patients with concomitant Sjogren’s syndrome.
- Other causes of xerostomia (i.e. pharmacological treatment).

### Interventions

A baseline visit (T0) was conducted 3 months after the end of RT. Demographic data and a thorough medical history were recorded with particular attention to oncologic history (i.e. chemotherapy, oncologic surgery, and RT dose) and smoking habits.

The basal and stimulated salivary flow rates were measured, and patients were asked to complete the questionnaires to assess their xerostomia grade. The unstimulated salivary flow rate was assessed using the spitting method. Patients were instructed to collect their saliva for 5 min in a graded tube. The stimulated salivary flow was determined in a similar manner. Salivary secretion was stimulated by applying a solution of 2% citric acid to the sides of the tongue at intervals of 30 s [18].

The investigational product sodium-hyaluronate mouthwash (Hydral**®**, Sunstar Italiana SRL. Saronno, VA, Italy) or the placebo was administered for a 30-day treatment period according to the allocation envelope. The placebo was made of water with added xylitol. Xylitol is a sugar alcohol used as a sugar substitute that does not increase the risk of tooth decay. In this formulation, its only role was to give a slightly sweet flavour to water. Water supplemented with xylitol, in fact, may reduce xerostomia symptoms to a certain extent, so it was considered a suitable comparator. An instruction procedure to standardise product administration was finalised. Patients were asked to rinse with 15 mL of the product using an appropriate dispenser three times a day.

**Fig. 1:**
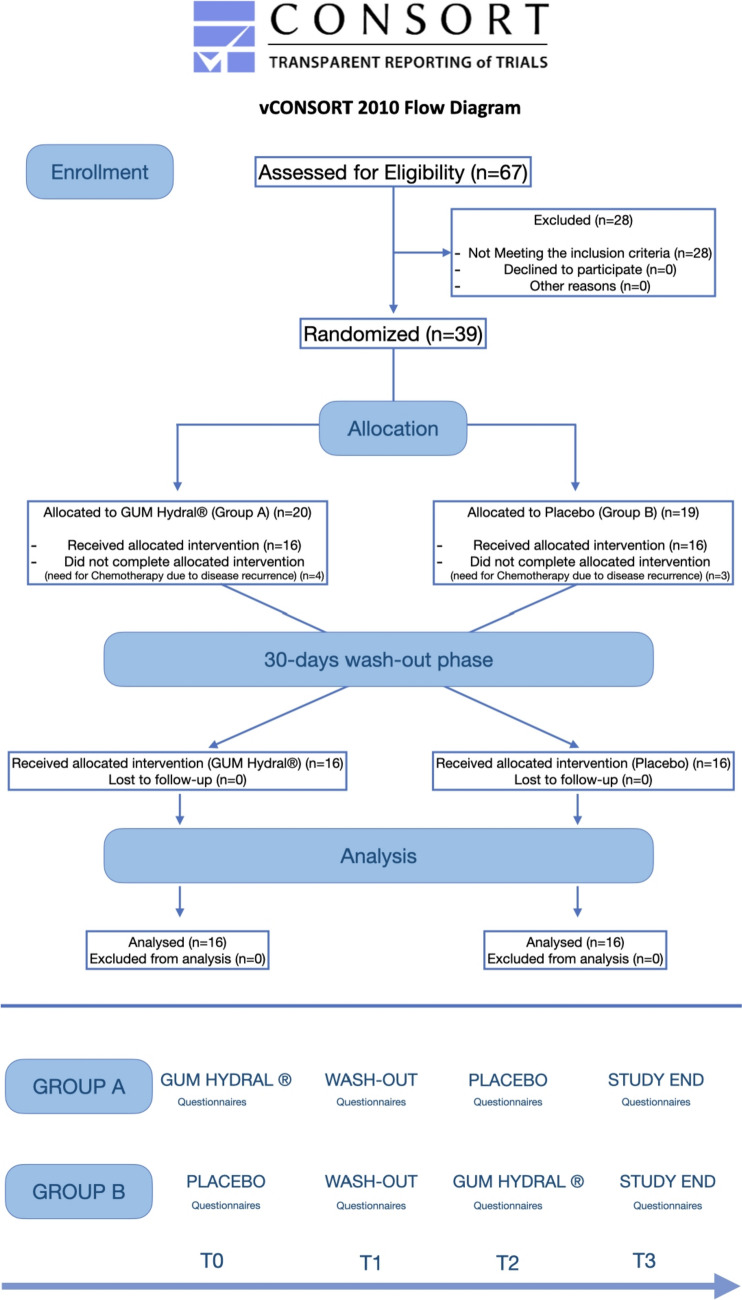
Consort flow diagram of the trial

The patients were divided into two groups: on day 1 (baseline—T0), after the questionnaires were answered, group A received the treatment product and group B received the placebo. On day 30, the administration of the products was stopped, and the questionnaires were administered together with the Likert scale. Following this, a 30-day washout phase was conducted, followed by Phase 2 of the study. On day 61, patients completed the questionnaires again, and each group was treated with the alternative product. On day 90, the final questionnaires were administered, and all endpoints were investigated. Each visit is described in the study flowchart (Fig. 1, CONSORT flow diagram, adapted from Marchetti et al. [19]). During every visit, a thorough clinical examination was performed, to control whether any clinical signs of adverse event could be noticed.

### Outcome measures

The primary objective of this RCT was to evaluate the reduction in patients’ xerostomia symptoms due to RT in contrast to hyposalivation, which can be objectively evaluated using sialometry. The measurement of xerostomia is problematic because it comprises a set of symptoms, and therefore can be assessed only by directly questioning individuals. The following questionnaires were used in this RCT: a modified version of the XQ [13, 20], the EORTC QLQ-C30 and QLQ-H&N35 questionnaires [16], and a Likert scale. The answers to the questionnaires were converted into a linear scoring scale, with values between 0 and 100, [21, 22] as advocated by the EORTC. Detailed information about the questionnaires as well as a copy of the questionnaires are available in the Supplementary Files (S2–S6).

### Randomisation sequence, allocation concealment, and blinding

The statistician and principal investigator (C.L.) generated the allocation sequence using a simple computer-generated random list. Allocation concealment was performed using sequentially numbered opaque sealed envelopes (SNOSE) [21]. Each patient was identified using a univocal code (SSHNC-1_n°). The treatment assignment was noted in a specific form maintained by the study registrar (C.R.), who also assigned the interventions to the patients. After the enrolment of each patient, the opaque envelope was opened, and the product was given to a masked coworker (A.B. or G.G.), who was responsible for enrolling the participants, administering the questionnaires and therapies to the patients, and visiting the patients throughout the entire duration of the study. Masking of the examiners was maintained throughout all experimental procedures, and both the study product and placebo were contained in identical bottles, differing only in a removable label. When assigning the treatment (during both Phases 1 and 2 of the study), the examiner received the bottles without the label, thus being unaware of the product. The product was then delivered to the patients who were blinded to the selected product.

### Sample size and statistical methods

Assuming that sodium-hyaluronate mouthwash can cause a 20% reduction in the XQ scores when compared with the placebo, setting a bilateral 95% confidence interval and a study power of 80% in a crossover design, a minimum sample of 32 is required. Assuming a dropout rate of 20%, a sample size of 40 patients was required. The calculation was carried out using PASS2021 software. Such a high dropout rate was decided because of the particularly fragile nature of HNC patients and difficulty in predicting the course of cancer therapy in these patients.

The clinical and demographic characteristics of the sample were described by applying descriptive statistical techniques. Qualitative variables are described using absolute and percentage frequencies, while quantitative variables are summarised either as mean and standard deviation (SD) when normally distributed or as median and interquartile range (IQR) when non-normally distributed. The intraclass correlation coefficient (ICC) test was used to determine the reliability between patients for each questionnaire at each time point. The ICC test was based on each question and the overall score. A coefficient major of 0.7 was considered reliable.

The normality of quantitative variables was assessed using the Shapiro–Wilk test. Differences between groups for quantitative variables were assessed by the Mann–Whitney *U* test if not normally distributed, or by the analysis of variance (ANOVA) test in the case of normally distributed variables. Differences in qualitative variables were assessed using the *χ*^2^ or Fisher’s exact test, as appropriate.

If any of the variables were associated with a reduction in xerostomia in univariate analysis, it was entered into a multivariate logistic regression model by inserting the therapy as a covariate and the XQ scores as outcome variables.

Statistical significance was set at *p* < 0.05. All statistical analyses were performed using a computer program (SPSS v 21.0, Chicago, NY, USA).

## Results

### Participant flow and recruitment

The patients participating in this study were recruited between October 2020 and March 2022; the last follow-up consultation was conducted 3 months later, in June 2022. Thirty-nine patients were recruited. Of these, seven patients dropped out during the treatment because of cancer recurrence and the need for chemotherapy (four from Group A and three from Group B). The results of the remaining 32 patients were used for statistical analysis. Further details regarding the study course are shown in Fig. 1.

### Baseline data and numbers analysed

The final sample included 32 patients (13 women and 19 men), with a mean age of 54.6 years (SD: 10.7; range: 24–72). The general patient characteristics are shown in Table 1.

**Table 1:** General characteristics of the included sample. No statistically significant differences were found between the groups

		Group A (16 patients)	Group B (16 patients)	Total sample (32 patients)
Gender	Men	12 (75%)	6 (37.5%)	19 (59.4%)
	Women	4 (25%)	10 (62.5%)	13 (40.6%)
Age	Mean	54.2 (27–64; SD: 10.4)	55.1 (24–72; SD: 10.8)	54.7 (24–72; SD: 10.7)
	< 40	2 (12.5%)	1 (6.2%)	3 (9.4%)
	40–49	2 (12.5%)	3 (18.8%)	5 (15.6%)
	50–59	4 (25%)	6 (37.5%)	10 (31.3%)
	60–69	8 (50%)	5 (31.3%)	13 (40.6%)
	70–79	0 (-)	1 (6.2%)	1 (3.1%)
Tumour type	Squamous cell carcinoma	15 (93.8%)	14 (87.5%)	29 (90.6%)
	Other types	1 (6.2%)	2 (12.5%)	3 (9.4%)
Tumour stage	Stage 1	1 (6.2%)	0 (-)	1 (3.1%)
	Stage 2	3 (18.8%)	1 (6.2%)	4 (12.5%)
	Stage 3	4 (25%)	6 (37.5%)	10 (31.3%)
	Stage 4	8 (50%)	9 (56.3%)	17 (53.1%)
Tumour site	Hipopharynx	2 (12.5%)	0 (-)	2 (6.3%)
	Larynx	2 (12.5%)	1 (6.2%)	3 (9.4%)
	Oral cavity	4 (25%)	4 (25%)	8 (25%)
	Oropharynx	1 (6.2%)	6 (37.5%)	7 (21.9%)
	Rhinopharynx	5 (31.3%)	1 (6.2%)	6 (18.8%)
	Salivary glands	1 (6.2%)	2 (12.5%)	3 (9.4%)
	Other sites	1 (6.2%)	2 (12.5%)	3 (9.4%)
Smoking	Smokers	8 (50%)	9 (56.3%)	17 (53.1%)
	No smokers	8 (50%)	7 (43.7%)	15 (46.9%)
Diabetes	Yes	1 (6.2%)	0 (-)	1 (3.1%)
	No	15 (93.8%)	16 (100%)	31 (96.9%)
Surgery	Performed	5 (31.3%)	8 (50%)	13 (40.6%)
	Not performed	11 (68.7%)	8 (50%)	19 (59.4%)
Chemotherapy	Performed	12 (75%)	8 (50%)	20 (62.5%)
	Not performed	4 (25%)	8 (50%)	12 (37.5%)
Unstimulated salivary flow	Mean (mL/5 min)	0.7 (0–1.5; SD: 0.5)	0.5 (0–1.5; SD: 0.5)	0.6 (0–1.5; SD: 0.5)
Stimulated salivary flow	Mean (mL/5 min)	1 (0–2; SD: 0.6)	0.85 (0–2; SD: 0.6)	0.92 (0–2; SD: 0.6)
RT dose	Mean (Gy)	68.8 (66–70; SD: 1.8)	65.8 (50–70; SD: 5.8)	67.3 (50–70; SD: 4.6)

The main results, as well as the scores recorded after the 1-month washout, were as follows: in Group A, the modified XQ score was 70.1 (SD: 25.7; range: 15.4–100) at baseline and 63.2 (SD: 24.3; range: 15.4–96.8) after the washout phase; for Group B patients, the modified XQ score was 58 (SD: 21.2; range:20.9–81.4) at baseline and 59.9 (SD: 21; range: 16.5–90.2) after the washout phase.

No statistically significant difference was found between any of the analysed variables (total and divided according to the different categories) or between the groups at different time points. Consequently, the baseline scores were grouped together with the scores after the washout phase, and then the statistical analyses were stratified according to the administered product.

To establish the reliability of patients’ answers to the questionnaires, an ICC analysis was performed between baseline questionnaires (before the beginning of Phase 1) and the questionnaires at the beginning of Phase 2, matching the answers for each patient to the same questions: ICC measured at baseline was 0.834, while that measured after the washout period was 0.866.

### Outcomes and estimation

Table 2 shows the overall effect of sodium-hyaluronate mouthwash and placebo in reducing the scores of the single questionnaires and the values recorded on the Likert scale after the treatment. Statistically significant lower values of the modified XQ were retrieved when comparing the baseline scores to those after treatment with the sodium-hyaluronate mouthwash: mean baseline score: 65 (SD: 23.6; 15.4–100), mean final score: 48.4 (SD: 24.1; 6.6–88, Mann–Whitney *U* test = 0.01). Statistically significant higher values of patient satisfaction (Likert scale) were retrieved for the sodium-hyaluronate mouthwash when compared with the placebo: mean Likert scale score for sodium-hyaluronate mouthwash: 64.1 (SD: 20.3; 15–100), mean Likert scale score for placebo: 31.9 (SD: 16.7; 15–60, Mann–Whitney *U* test = 0.001). Statistically significant improvements in the values of the modified XQ were obtained when comparing the treatment with sodium-hyaluronate mouthwash (16.7; SD: 11.3, 9.9–41.8) with the placebo treatment (4.9; SD: 13, 10.3–57.2, Mann–Whitney *U* test = 0.001).

**Table 2:** Differences in the scores for each questionnaire after the two different treatments—every score was converted in a linear value (range: 1–100)

	Modified XQ ^a b^	QLQ-C30						QLQ-H&N35							Likert scale ^e^
	Total	Overall	Physical	Role	Emotional	Social	Total	Pain	Swallowing	Functional and sensorial	Eating	Social	Speech	Total	
Baseline scores: *mean (standard deviation; min–max) *^*c, d*^															
Sodium-hyaluronate mouthwash	65 (23.6; 15.4–100)	28.6 (13.3; 0–64.3)	38.9 (11.6; 25–75)	35.4 (12.2; 25–68.75)	39.9 (14.9; 25–83.3)	40.4 (14.2; 25–75)	37.7 (10.3; 23.8–60.3)	37.4 (11.2; 27.3–72.7)	46.9 (12.1; 25–70.8)	41.9 (18.4; 25–94.2)	51.8 (12.1; 37.5–81.3)	34.6 (13.6; 22.2–75)	39.5 (19.3; 25–87.5)	41.1 (10.1; 26.9–71.5)	
Placebo	60.6 (22.6; 15.4–96.8)	25.7 (11.8; 0–42.9)	36.7 (8.7; 18.3–58.3)	36.1 (11.2; 25–62.5)	36.7 (13.3; 20.8–70.8)	35.9 (12.1; 25–58.3)	35.3 (8.2; 21.4–51.6)	35.8 (10.1; 22.7–63.6)	45.4 (15.9; 29.2–100)	46.4 (14.4; 29.2–87.5)	50.4 (13.1; 31.3–81.3)	33.3 (15.6; 8.3–88.9)	40.6 (19.1; 25–75)	40.9 (10.5; 29.2–78.5)	
Scores after treatment: *mean (standard deviation; min–max)*															
Sodium-hyaluronate mouthwash ^a e^	48.4 (24.1; 6.6–88)	27.7 (15; 0–57.1)	35.8 (8.7; 25–58.3)	34.6 (11.3; 25–56.3)	37.4 (13.7; 25–70.8)	37.8 (15.4; 25–75)	35.2 (9.3; 22.2–56.4)	35.5 (10.8; 27.3–72.7)	44 (11.9; 25–70.8)	41.4 (13.4; 25–75)	50.2 (10.7; 37.5–75)	34.6 (11.9; 25–63.9)	39.1 (18.7; 25–87.5)	39.9 (9.6; 26.9–71.5)	64.1 (20.3; 15–100)
Placebo ^f^	55.7 (23.9; 14.3–94.6)	28.1 (12.8; 0–57.1)	35.4 (8.4;18.3–51.7)	34.2 (11.3; 25–68.75)	36.2 (11.3; 20.8–66.7)	35.4 (12.5; 25–66.7)	34.6 (7.5; 21.4–50.8)	34.8 (7.9; 27.3–50)	43.2 (12.1; 20.8–66.7)	41.9 (12.3; 25–66.7)	49 (11.7; 31.3–75)	31.9 (11.9; 16.7–66.7)	39.5 (17.4; 25–87.5)	38.9 (7.4; 25.4–53.8)	31.9 (16.7; 15–60)
Reduction of the scores after the treatment: *mean (standard deviation; min–max)*															
Sodium-hyaluronate mouthwash ^b^	16.7 (11.3; − 9.9–41.8)	0.9 (10.9; − 35.7–21.4)	3.1 (5.9; − 6.7–26.7)	0.8 (7.1; − 18.8–18.8)	2.6 (10.3; − 33.3–20.8)	2.6 (11.1; − 33.3–20.8)	2.4 (4.1; − 7.1–8.7)	1.8 (8.8; − 27.3– 18.2)	2.9 (7.1; − 8.3–20.8)	0.5 (9.7; − 16.7 41.7)	1.6 (6; − 12.5–18.8)	− 0.1 (6.7; − 19.4–22.2)	0.4 (16; − 50–37.5)	1.1 (3.8; − 7.7–10)	
Placebo	4.9 (13; − 10.3 – 57.2)	− 2.5 (11.4; − 28.6–28.6)	1.3 (3.9; − 3.3–11.7)	1.9 (7.4; − 12.5–25)	0.5 (8.2; − 16.7–25)	0.5 (8.2; − 16.7–25)	0.7 (3.6; − 7.1–7.9)	1 (7.3; − 13.6– 18.2)	2.2 (14.5; − 25–41.7)	4.4 (11.2; − 12.5–41.7)	1.4 (9.5; − 25–18.8)	1.3 (16.5; − 44.4–72.2)	1.2 (8.6; − 12.5–25)	2 (8.2; − 13.1–32.3)	

^a^Statistically significantly lower values of the modified XQ were retrieved when comparing the baseline scores to the ones after treatment with sodium-hyaluronate mouthwash (Mann**–**Whitney *U* test = 0.01)

^b^Statistically significantly higher improvement in the values of the modified XQ was retrieved when comparing the treatment with sodium-hyaluronate mouthwash to the placebo (Mann**–**Whitney *U* test = 0.001)

^c^No statistically significant difference was retrieved between groups A and B when comparing the baseline scores of the questionnaire (Mann**–**Whitney *U* test > 0.05)

^d^No statistically significant difference was retrieved between placebo and sodium-hyaluronate mouthwash groups when comparing the baseline scores of the questionnaire (Mann**–**Whitney *U* test > 0.05)

^e^Statistically significantly higher values of patient’s satisfaction (Likert scale) were retrieved for the sodium-hyaluronate mouthwash (Mann**–**Whitney *U* test = 0.001)

^f^No statistically significant difference was retrieved between the two groups when comparing the baseline scores to the ones after treatment with placebo mouthwash (Mann**–**Whitney *U* test > 0.05)

Furthermore, no adverse events due to the use of sodium-hyaluronate mouthwash were noticed in any of the included patients.

## Discussion

RT may cause significant adverse events in the oral cavity of patients with HNC (i.e. osteoradionecrosis, dysphagia, dysgeusia, caries, and trismus) [5–7]. RIX is one of the most severe consequences of RT and significantly affects patients’ QoL. The absence of salivary flow may reduce the clearance of oral fluids by bacteria, resulting in oral environment changes (i.e. chronic candidiasis), tooth demineralisation and rampant caries, mucosal dehydration and atrophy, and reduced lubrication of mucosal tissues, which may result in difficulty in chewing, speaking, and swallowing [23]. Although the introduction of intensity-modulated radiation therapy (IMRT) enables RT to spare a large volume of salivary glands and being selective for cancer [24], and some preventive measures have been proposed (e.g. amifostine therapy [25]), salivary gland hypofunction is a common and permanent adverse effect that cannot be avoided. Several studies have attempted to improve the management of RIX, but its treatment remains symptomatic [26]. A therapeutic option may be based on pilocarpine and other muscarinic agonists (e.g. cevimeline) to slightly improve symptomatology [27]. Nevertheless, these medications do not prevent salivary gland damage and cannot be prescribed in cases of heart failure or other cardiovascular diseases [28, 29]. Consequently, in several cases of irreversible damage to the salivary parenchyma, salivary substitutes may be the only available treatment option [10]. A wide variety of salivary substitutes are commercially available based on different active substances (i.e. carboxymethylcellulose, xanthan gum, herbal compounds) in several forms (i.e. sprays, gels, and intraoral devices for slow release) [30]. However, very weak evidence demonstrating the superiority of a specific product in terms of beneficial effects on symptom relief is available [31]. The aim of this study was to evaluate the reduction in patients’ xerostomia symptoms caused by RT after the use of sodium-hyaluronate mouthwash. The main results of this RCT show how sodium-hyaluronate mouthwash can be effective in reducing the burden of RIX symptoms; in particular, a statistically significant improvement in the values of the modified XQ was observed when comparing sodium-hyaluronate mouthwash treatment with placebo treatment. Furthermore, no statistically significant difference emerged between the results of the questionnaires at baseline and those submitted to the patients at the end of the washout period. This means that the washout period was adequately observed and cancelled out the effects attributable to the administration of the first product. This finding is particularly relevant because it does not invalidate the reliability of the crossover study design. Despite the fact that these results are particularly interesting and statistically significant, the results also demonstrated that although both QLQ-C30 and QLQ-H&N35 showed an overall improvement when compared with the baseline, the results of the statistical analysis were not statistically significant for either of the two products. These findings can be explained by the fact that the modified XQ has one specific field of investigation, and its questions are more focused on RIX. However, the QLQ-C30 and QLQ-H&N35 investigated several aspects of QoL: the QLQ-C30 globally assesses the patients’ QoL, while the QLQ-H&N35 addresses symptoms associated with the specific tumour location or its treatment. QLQ-H&N35 did not show a higher improvement when compared with QLQ-C30. This may be explained by the fact that, although QLQ-H&N35 is more centred on the local symptoms, HNC patients may suffer from a high number of local alterations (i.e., osteoradionecrosis, trismus, dental caries), which may have a greater impact on their answers to the QoL questionnaires, when compared with RIX. HNC patients are a particularly challenging study sample as indicated by the high number of dropouts. Seven patients had to discontinue their participation in the protocol because of the worsening of the oncological pathology that necessitated the start of new chemotherapy cycles. It was not possible to perform an intention-to-treat analysis, as all patients discontinued the first two treatments in the study. However, the possibility that the number of dropouts in this cohort of patients would be high was already anticipated when calculating the sample size; therefore, the power of the study was not impaired. Extensive statistical work was performed to assess whether anamnestic variables influenced the modified XQ, but none of them reached statistical significance (Mann–Whitney *U* test, *p* > 0.05). Furthermore, although the patients included were heterogeneous in terms of baseline tumour and overall QoL, the included sample showed great homogeneity for the main variables; in particular, all the patients were treated with high-dose RT (mean 67.3 Gy) and showed severe hyposalivation, thus making it difficult to retrieve a specific salivary flow amount that could be used as a cut-off value linked to an improvement. Similarly, it was impossible to state whether a lower RT dose (i.e. palliative RT) could be related to a higher relief in symptoms. It has been demonstrated that the habit of smoking can reduce the salivary flow [32], but its influence on a salivary substitute therapy has never been evaluated. Furthermore, the severity of RIX could have been influenced by a large time interval between the end of RT and the enrolment in this study. In fact, it has been demonstrated that RIX may worsen up to 18 months after the end of RT [11]. Nevertheless, no statistical differences were found between the groups at baseline, and, furthermore, the placebo/crossover design could have mitigated for this factor, thus making the risk for this specific bias negligible.

No differences were found in the other variables. Probably, RT has a significant damaging effect on the salivary glands, making the effect of smoking negligible. The same explanation could be provided regarding previous oncologic therapies (chemotherapy and surgery), which did not show any influence on xerostomia symptoms, confirming how RIX is mainly or totally due to RT damage to the salivary glands. Finally, this study did not highlight any differences in patient age. The incidence of xerostomia is usually higher in older patients because they show several comorbidities and undergo pharmacological therapies, which usually lead to hyposalivation and xerostomia [33]. A possible explanation is that our study excluded the presence of pharmacological treatment, which could be associated with a reduction in salivary flow as an exclusion criterion. An interesting issue is that the results of this trial are difficult to compare with those of previous studies on salivary substitutes since only a few have reported quantitative results. Stewart et al. compared three commonly used categories of non-prescription products in 80 patients: sorbitol-sweetened sour lemon lozenge, a sorbitol/xylitol-sweetened chewing gum, and sorbitol/xylitol-sweetened artificial saliva substitute spray. Each product provided brief symptomatic relief from xerostomia, but no product was superior to the others. Unfortunately, the authors administered a visual analog scale rating the oral dryness only after the use of each product, not providing any comparison between the baseline and the end of the treatment, making a comparison with our results impossible [34]. McMillan et al. tested a sorbitol-based gel in 22 patients in a randomised single-blind crossover study. The authors registered a decrease of 4% in the xerostomia inventory after using the product for 4 weeks. Our study is in line with this result but reports a slightly higher improvement [35]. Barbe et al. (2018) [15] compared the efficacy of sodium-hyaluronate mouthwash (Hydral**®**, Sunstar Italiana SRL. Saronno, VA, Italy) and the above-mentioned gel made of sorbitol in a randomised clinical trial. They showed that both products can reduce xerostomia symptoms (*p* < 0.05), but a comparison with the present study is impossible since a quantitative evaluation of the improvement of the symptoms is not provided in their study. Marimuthu et al. (2020) [36] tested an immunologically active salivary substitute in a prospective, double-blind, randomised, placebo-controlled study on irradiated nasopharyngeal HNC patients. The included patients showed a substantial improvement in their RIX (25% of the XI score) compared with placebo (16% improvement). Our results showed a lower reduction of RIX symptoms. Probably, the patients included in Marimuthu’s RCT suffered from a less severe RIX, when compared with the ones included in the present study, because of the absence of anatomical contiguity between the irradiated field (nasopharynx) and the salivary glands, allowing a better performance of the salivary substitute therapy, as confirmed by the good performance for the control group also. Marin et al. (2021) compared two sodium fluoride-based salivary substitutes with a placebo spray [37]. All included products (including the placebo) showed statistically significant efficacy in reducing RIX symptoms. Nevertheless, the authors only evaluated the efficacy of the products using the VAS scale. Furthermore, it could be highlighted that no irradiated patients were included in this study, making the population very different from that analysed in the present study. This study has several strengths. All the included patients had been irradiated at the same centre (hence the same machine and therapy protocol), which, together with the crossover design, helped mitigate the potential confounding effect caused by the heterogeneity of the different HNCs. Nevertheless, the crossover design has another advantage. The inter-individual variability that may be found in questionnaire-based studies investigating PROMS is greatly mitigated by the fact that each patient was his or her own control, strongly reducing the allocation bias. In conclusion, the results of the study are encouraging, since no adverse events due to the use of sodium-hyaluronate mouthwash were noticed, and the product led to a subjective improvement in the sensation of dry mouth, as shown by the modified XQ. In fact, given the lack of effective RIX treatments, non-invasive treatment based on salivary substitutes may represent the main alternative to improve xerostomia symptoms and, consequently, the overall QoL of patients. The product is also quite inexpensive, considering the average cost in Italy (between 5 and 10 euros per bottle, where two bottles are sufficient for a month), a cost that is hypothetically sustainable even by the National Health System. Nevertheless, this study also had some limitations. Although sodium-hyaluronate mouthwash determined a significant improvement in RIX symptoms, a 16% improvement still provides a partial benefit, which is sufficient to significantly change the overall QoL of the patients. Furthermore, statistical analysis failed to reveal whether a specific category of HNC patients would benefit more or less from the therapy. Future studies are needed to understand whether more targeted therapies based on salivary replacement are indicated.

Another limitation of this study is that we only enrolled 32 patients. Although the crossover design has virtually allowed for doubling the sample size, further trials, with more patients, are needed to increase the generalisability of our results.

In conclusion, the product has shown a promising effect in reducing the discomfort associated with RIX, but other potential effects, such as its role in preventing caries and modifying the oral microbiota by reducing the risk of fungal infections, are yet to be determined in further trials.

## Conclusions

The results of this double-blind RCT highlight the advantages of treating RIX with sodium-hyaluronate mouthwash, as it seems to be clinically effective in offering relief of RIX symptoms in the majority of the patients, without any reported adverse events.

### Supplementary Information

Below is the link to the electronic supplementary material.

Supplementary file1 (DOC 219 KB)

Supplementary file2 (DOCX 17 KB)

Supplementary file3 (DOCX 35 KB)

Supplementary file4 (PDF 41 KB)

Supplementary file5 (PDF 105 KB)

Supplementary file6 (DOCX 13 KB)

## Data Availability

Data are available upon request to the authors.

## References

[1] Sung H, Ferlay J, Siegel RL, Laversanne M, Soerjomataram I, Jemal A, Bray F (2021). Global Cancer Statistics 2020: GLOBOCAN estimates of incidence and mortality worldwide for 36 cancers in 185 countries. CA Cancer J Clin.

[2] Liu J, Kaplon A, Blackman E, Miyamoto C, Savior D, Ragin C (2020). The impact of the multidisciplinary tumor board on head and neck cancer outcomes. Laryngoscope.

[3] Berrone M, Lajolo C, De Corso E (2021). Cooperation between ENT surgeon and dentist in head and neck oncology. Acta Otorhinolaryngol Ital.

[4] Marur S, Forastiere A (2016). Head and neck squamous cell carcinoma: update on epidemiology, diagnosis, and treatment. Mayo Clin Proc.

[5] Lajolo C, Gioco G, Rupe C (2021). Tooth extraction before radiotherapy is a risk factor for developing osteoradionecrosis of the jaws: a systematic review. Oral Dis.

[6] Lajolo C, Rupe C, Gioco G (2021). Osteoradionecrosis of the jaws due to teeth extractions during and after radiotherapy: a systematic review. Cancers.

[7] Massaccesi M et al (2022) A predictive nomogram for trismus after radiotherapy for head and neck cancer. Radiother Oncol 2:S0167–8140(22)04137–8. 10.1016/j.radonc.2022.05.031 35662658

[8] Rupe C, Gioco G, Almadori G (2022). Oral Candida spp. Colonisation is a risk factor for severe oral mucositis in patients undergoing radiotherapy for head & neck cancer: results from a multidisciplinary mono-institutional prospective observational study. Cancers.

[9] Assas M, Wiriyakijja P, Fedele S, Porter S, Ní RR (2021). Evaluating the measurement properties of patient-reported outcome measures in radiotherapy-induced xerostomia. Oral Dis.

[10] Mercadante V, Al Hamad A, Lodi G, Porter S, Fedele S (2017) Interventions for the management of radiotherapy-induced xerostomia and hyposalivation: a systematic review and meta-analysis. Oral Oncol 10.1016/j.oraloncology.2016.12.031 28249650

[11] Sroussi H, Epstein J, Bensadoun R (2017). Common oral complications of head and neck cancer radiation therapy: mucositis, infections, saliva change, fibrosis, sensory dysfunctions, dental caries, periodontal disease, and osteoradionecrosis. Cancer Med.

[12] Contaldo M et al (2022) Oral candidiasis and novel therapeutic strategies: antifungals, phytotherapy, probiotics, and photodynamic therapy. Curr Drug Deliv 18 10.2174/1567201819666220418104042 35440307

[13] Memtsa P, Tolia M, Tzitzikas I et al (2017) Assessment of xerostomia and its impact on quality of life in head and neck cancer patients undergoing radiation therapy. Mol Clin Oncol 10.3892/mco.2017.1200 PMC5431738 28529753

[14] Kang M, Park H, Jun J, Son M, Kang M (2017) Facilitated saliva secretion and reduced oral inflammation by a novel artificial saliva system in the treatment of salivary hypofunction. Drug Des Devel Ther 10.2147/DDDT.S121254 PMC5241125 28138222

[15] Barbe A, Schmidt-Park Y, Hamacher S, Derman S, Noack M (2018) Efficacy of GUM® Hydral versus Biotène® Oralbalance mouthwashes plus gels on symptoms of medication-induced xerostomia: a randomized, double-blind, crossover study. Clin Oral Investig 10.1007/s00784-017-2096-0 28353023

[16] Pilz M, Gamper E, Efficace F, et al. EORTC QLQ-C30 general population normative data for Italy by sex, age and health condition: an analysis of 1,036 individuals. BMC Public Health(22 ):1040. 10.1186/s12889-022-13211-y PMC9128281 35610611

[17] Dwan K, Altman TLD, Elbourne D (2019). CONSORT 2010 statement: extension to randomised crossover trials. BMJ.

[18] Villa A, Connell C, Abati S (2014) Diagnosis and management of xerostomia and hyposalivation. Ther Clin Risk Manag 10.2147/TCRM.S76282 PMC4278738 25653532

[19] Marchetti E, Mummolo S, Di Mattia J (2011). Efficacy of essential oil mouthwash with and without alcohol: a 3-day plaque accumulation model. Trials.

[20] Thomson W, Jane M, Chalmers J, Spencer S, Williams M (1999) The Xerostomia inventory: a multi-item approach to measuring dry mouth. Commun Dental Health 10697349

[21] Bjordal K, Hammerlid E, Ahlner-Elmqvist M (1999). Quality of life in head and neck cancer patients: validation of the European Organization for Research and Treatment of Cancer Quality of Life Questionnaire-H&N35. J Clin Oncol.

[22] Aaronson N, Ahmedzai S, Bergman B (1993). The European Organization for Research and Treatment of Cancer QLQ-C30: a quality-of-life instrument for use in international clinical trials in oncology. J Natl Cancer Inst.

[23] Epstein J, Stevenson-Moore P (1992). A clinical comparative trial of saliva substitutes in radiation-induced salivary gland hypofunction. Spec Care Dentist.

[24] Gomez-Millan J, Fernández J, Medina CJ (2013). Current status of IMRT in head and neck cancer. Rep Pract Oncol Radiother.

[25] Bourhis J, Rosine D (2002). Radioprotective effect of amifostine in patients with head and neck squamous cell car-cinoma. Semin Oncol.

[26] Pinna R, Campus G, Cumbo E, Mura I, Milia E (2015). Xerostomia induced by radiotherapy: an overview of the physiopathology, clinical evidence, and management of the oral damage. Ther Clin Risk Manag.

[27] Mercadante V, Jensen S, Smith D (2021). Salivary gland hypofunction and/or xerostomia induced by nonsurgical cancer therapies: ISOO/MASCC/ASCO guideline. J Clin Oncol.

[28] Nagele P, Rao L, Penta M (2011). Postoperative myocardial injury after major head and neck cancer surgery. Head Neck.

[29] Ramírez M, Hernández R, Casañas E, Serrano J, Hernández G, López-Pintor R (2020). Xerostomia and salivary flow in patients taking antihypertensive drugs. Int J Environ Res Public Health.

[30] Ameri A, Heydarirad G, Rezaeizadeh H, Choopani R, Ghobadi A, Gachkar L (2016) Evaluation of efficacy of an herbal compound on dry mouth in patients with head and neck cancers: a randomized clinical trial. J Evid Based Complementary Altern Med 10.1177/2156587215590232 26137850

[31] Assery M (2019) Efficacy of artificial salivary substitutes in treatment of xerostomia: a systematic review. J Pharm Bioallied Sci 11 10.4103/jpbs.JPBS_220_18 PMC6398314 30923424

[32] Rad M, Kakoie S, Niliye Brojeni F, Pourdamghan N (2010). Effect of long-term smoking on whole-mouth salivary flow rate and oral health. J Dent Res Dent Clin Dent Prospects.

[33] Gil-Montoya J, Silvestre F, Barrios R, Silvestre-Rangil J (2016). Treatment of xerostomia and hyposalivation in the elderly: a systematic review. Med Oral Patol Oral Cir Bucal.

[34] Stewart C, Jones A, Bates R, Sandow P, Pink F, Stillwell J (1998). Comparison between saliva stimulants and a saliva substitute in patients with xerostomia and hyposalivation. Spec Care Dentist.

[35] McMillan A, Tsang C, Wong M, Kam A (2006). Efficacy of a novel lubricating system in the management of radi-otherapy-related xerostomia. Oral Oncol.

[36] Marimuthu D, Han K, Mohamad M (2021). Saliva substitute mouthwash in nasopharyngeal cancer survivors with xerostomia: a randomized controlled trial. Clin Oral Investig.

[37] Marín C, Díaz-de-Valdés L, Conejeros C, Martínez R, Niklander S (2021). Interventions for the treatment of xerostomia: a randomized controlled clinical trial. J Clin Exp Dent.

