# Supplementary material for: Sodium-hyaluronate mouthwash on radiotherapy-induced xerostomia: a randomised clinical trial
Source: Support Care Cancer. 2023 Oct 18;31(11):644. doi: 10.1007/s00520-023-08090-x (PMC10584731; doi:10.1007/s00520-023-08090-x)
Supplement: Supplementary file 2 — Supplementary file2 (DOCX 17 KB) [file 520_2023_8090_MOESM2_ESM.docx]

PATIENTS-ADMINISTERED QUESTIONNAIRES

Xerostomia Questionnaire (XQ) provides a measure of the severity of radiation-induced xerostomia that affects the patients' QoL. This questionnaire consists of 9 questions, associated with patient-reported dryness. The. XQ was modified as follows: one further question was added from Xerostomia Inventory (XI) scale score. Moreover, a general question “how dry is your mouth?” was added to the questionnaire. Thus, the modified XQ provides a measure of the severity of radiation -induced xerostomia that affects the patients' QoL. This questionnaire consists of 11 questions, associated with patient-reported dryness. The modified XQ is a self-administered tool and patients were asked to rate each symptom on a 10-point visual number scale (NRS) of 0-10, with higher scores indicating more severe dryness or discomfort due to dryness. Each item score is added, and the sum is linearly transformed to produce the final summary score ranging from 0 to 100, with higher scores representing higher levels of xerostomia – see appendices for all the questionnaires.

The secondary objectives of this study were: (1) to evaluate the improvement of QoL, throughout the EORTC QLQ-C30 and QLQ-H&N35 questionnaires [15]: the QLQ-C30 provides 30 questions, globally assessing patients’ quality of life. The QLQ-C30 questions can be grouped in five areas of interest: the overall health condition of the patient, the physical issues, role functions, emotional and social issues [12]. The QLQ-H&N35 includes 35 questions, and addresses symptoms associated with specific tumor location, side effects associated with the oncologic treatment and additional QoL aspects modified by the disease or its treatment. Of these 35 questions, 30 foresee an answer on a scale from 1 to 4, while 5 are simple answer questions. This questionnaire evaluates six categories of interest: pain (which comprises more specific questions related to the oral cavity district, differently from the category “physical issues” of QLQ-C30), swallowing issues, function, senses alteration, eating issues, speech issues and social issues. The last category mentioned in this questionnaire focuses on social eating and sexuality. The answers were converted into a linear scoring scale, with values between 0 and 100, [19] [20] as advocated by the European Organisation for Research and Treatment of Cancer (EORTC); (2) to evaluate the patients’ satisfaction in using the product throughout the use of a Likert scale: this further questionnaire consisted of 5 questions, which answers had a score ranging from 0 to 4: higher scores represented higher levels of satisfaction in using the product.
