# Supplementary material for: Sodium-hyaluronate mouthwash on radiotherapy-induced xerostomia: a randomised clinical trial
Source: Support Care Cancer. 2023 Oct 18;31(11):644. doi: 10.1007/s00520-023-08090-x (PMC10584731; doi:10.1007/s00520-023-08090-x)
Supplement: Supplementary file 3 — Supplementary file3 (DOCX 35 KB) [file 520_2023_8090_MOESM3_ESM.docx]

**Questionario di valutazione della xerostomia**

Iniziali del paziente ____________ Data __________

Gentile paziente, sono riportate in questo documento diverse domande che la aiuteranno a descrivere la secchezza nella sua bocca e il modo in cui tale secchezza modifica la sua vita quotidiana. Per favore, indichi con un cerchio il numero che corrisponde alla sua condizione durante l’ultima settimana, per ciascuna delle seguenti domande.

| **Esempio:**  Se la sua bocca è secca in alcune fasi del giorno (per esempio solo di notte), dovrebbe cerchiare “5”  Se la sua bocca è secca soltanto in alcune occasioni, come dopo aver compiuto uno sforzo fisico, dovrebbe cerchiare “3”  0 1 2 3 4 5 6 7 8 9 10  Per nulla Estremamente secco  secco |
| --- |

- Quanto è secca la sua bocca?

0 1 2 3 4 5 6 7 8 9 10

Per nulla secca Estremamente secca

1. Attribuisca un valore alla scomodità della sua protesi rimovibile a causa della secchezza (se non utilizza protesi, non compilare questo campo):

0 1 2 3 4 5 6 7 8 9 10

Comoda Estremamente scomoda

1. Attribuisca un valore alla sua difficoltà nel parlare a causa della secchezza della sua bocca e della sua lingua:

0 1 2 3 4 5 6 7 8 9 10

Facile Estremamente difficile

1. Attribuisca un valore alla sua difficoltà nel masticare il cibo a causa della secchezza:

0 1 2 3 4 5 6 7 8 9 10

Facile Estremamente difficile

1. Attribuisca un valore alla sua difficoltà nell’ingoiare il cibo a causa della secchezza:

0 1 2 3 4 5 6 7 8 9 10

Facile Estremamente difficile

1. Attribuisca un valore alla secchezza della sua bocca quando consuma un pasto:

0 1 2 3 4 5 6 7 8 9 10

Per nulla secca Estremamente secca

1. Attribuisca un valore alla secchezza della sua bocca quando non sta mangiando o masticando:

0 1 2 3 4 5 6 7 8 9 10

Per nulla secca Estremamente secca

1. Attribuisca un valore alla frequenza con cui utilizza liquidi per favorire la deglutizione del cibo:

0 1 2 3 4 5 6 7 8 9 10

Mai Estremamente frequente

1. Attribuisca un valore alla frequenza con cui utilizza liquidi per migliorare il benessere della bocca quando non mangia:

0 1 2 3 4 5 6 7 8 9 10

Mai Estremamente frequente

1. Attribuisca un valore alla frequenza dei suoi problemi durante il sonno a causa della secchezza della bocca:

0 1 2 3 4 5 6 7 8 9 10

Mai Estremamente frequenti

1. Attribuisca un valore alla sensazione di secchezza delle sue labbra

0 1 2 3 4 5 6 7 8 9 10

Per nulla secche Estremamente secche

* Da 1 a 9, originale di A. Eisbruch, traduzione e adattamento all’italiano a cura di C. Lajolo

** N. 10, originale di Thomson WM, traduzione e adattamento all’italiano a cura di C. Lajolo
