# Supplementary material for: Sodium-hyaluronate mouthwash on radiotherapy-induced xerostomia: a randomised clinical trial
Source: Support Care Cancer. 2023 Oct 18;31(11):644. doi: 10.1007/s00520-023-08090-x (PMC10584731; doi:10.1007/s00520-023-08090-x)
Supplement: Supplementary file 4 — Supplementary file4 (PDF 41 KB) [file 520_2023_8090_MOESM4_ESM.pdf]

EORTC QLQ-C30 (versione 3)

Con questo questionario vorremmo sapere alcune cose su di Lei e sulla Sua salute. La preghiamo di rispondere a tutte le domande ponendo un cerchio attorno al numero che meglio corrisponde alla Sua risposta. Non esiste una risposta "giusta" o "sbagliata". Le Sue informazioni verranno tenute strettamente riservate.

Per favore scriva solo le iniziali del Suo nome e cognome: |_|_|_|_|
Data di nascita (g, m, a): |_|_|_|_|_|_|_|_|
La data di oggi (g, m, a): 31 |_|_|_|_|_|_|_|_|

	No	Un po'	Parec-chio	Moltis-simo
1. Ha difficoltà nel fare lavori faticosi, come portare una borsa della spesa pesante o una valigia?	1	2	3	4
2. Ha difficoltà nel fare una lunga passeggiata?	1	2	3	4
3. Ha difficoltà nel fare una breve passeggiata fuori casa?	1	2	3	4
4. Ha bisogno di stare a letto o su una sedia durante il giorno?	1	2	3	4
5. Ha bisogno di aiuto per mangiare, vestirsi, lavarsi o andare in bagno?	1	2	3	4

Durante gli ultimi sette giorni:

	No	Un po'	Parec-chio	Moltis-simo
6. Ha avuto limitazioni nel fare il Suo lavoro o i lavori di casa?	1	2	3	4
7. Ha avuto limitazioni nel praticare i Suoi passatempi-hobby o altre attività di divertimento o svago?	1	2	3	4
8. Le è mancato il fiato?	1	2	3	4
9. Ha avuto dolore?	1	2	3	4
10. Ha avuto bisogno di riposo?	1	2	3	4
11. Ha avuto difficoltà a dormire?	1	2	3	4
12. Ha sentito debolezza?	1	2	3	4
13. Le è mancato l'appetito?	1	2	3	4
14. Ha avuto un senso di nausea?	1	2	3	4
15. Ha vomitato?	1	2	3	4
16. Ha avuto problemi di stitichezza?	1	2	3	4

Continuare alla pagina successiva

**Durante gli ultimi sette giorni:**	**No**	**Un po'**	**Parec-chio**	**Moltis-simo**
17. Ha avuto problemi di diarrea?	1	2	3	4
18. Ha sentito stanchezza?	1	2	3	4
19. Il dolore ha interferito con le Sue attività quotidiane?	1	2	3	4
20. Ha avuto difficoltà a concentrarsi su cose come leggere un giornale o guardare la televisione?	1	2	3	4
21. Si è sentito(a) teso(a)?	1	2	3	4
22. Ha avuto preoccupazioni?	1	2	3	4
23. Ha avuto manifestazioni di irritabilità?	1	2	3	4
24. Ha avvertito uno stato di depressione?	1	2	3	4
25. Ha avuto difficoltà a ricordare le cose?	1	2	3	4
26. Le Sue condizioni fisiche o il Suo trattamento medico hanno interferito con la Sua vita familiare?	1	2	3	4
27. Le Sue condizioni fisiche o il Suo trattamento medico hanno interferito con le Sue attività sociali?	1	2	3	4
28. Le Sue condizioni fisiche o il Suo trattamento medico Le hanno causato difficoltà finanziarie?	1	2	3	4

Per le seguenti domande ponga un cerchio intorno al numero da 1 a 7 che meglio corrisponde alla Sua risposta

29. Come valuterebbe in generale la Sua salute durante gli ultimi sette giorni?

1 2 3 4 5 6 7

Pessima Ottima

30. Come valuterebbe in generale la Sua qualità di vita durante gli ultimi sette giorni?

1 2 3 4 5 6 7

Pessima Ottima

© QLQ-C30 Copyright 1995 EORTC Quality of Life Group. Tutti i diritti sono riservati. Versione 3.0
