# Supplementary material for: Sodium-hyaluronate mouthwash on radiotherapy-induced xerostomia: a randomised clinical trial
Source: Support Care Cancer. 2023 Oct 18;31(11):644. doi: 10.1007/s00520-023-08090-x (PMC10584731; doi:10.1007/s00520-023-08090-x)
Supplement: Supplementary file 5 — Supplementary file5 (PDF 105 KB) [file 520_2023_8090_MOESM5_ESM.pdf]

# EORTC QLQ - H&N35

Talvolta i pazienti accusano i seguenti sintomi. La preghiamo di indicare il grado con cui ha provato questi sintomi durante gli ultimi sette giorni. Risponda tracciando un cerchio intorno al numero che meglio definisce la sua situazione.

---

| **Durante gli ultimi sette giorni:** | | **No** | **Un po'** | **Parec-chio** | **Moltis-simo** |
|---|---|---|---|---|---|
| 31. | Ha avuto dolore alla bocca? | 1 | 2 | 3 | 4 |
| 32. | Ha avuto dolore alla mascella? | 1 | 2 | 3 | 4 |
| 33. | Ha avuto infiammazione in bocca? | 1 | 2 | 3 | 4 |
| 34. | Ha avuto dolore alla gola? | 1 | 2 | 3 | 4 |
| 35. | Ha avuto problemi a deglutire liquidi? | 1 | 2 | 3 | 4 |
| 36. | Ha avuto problemi a deglutire cibi passati? | 1 | 2 | 3 | 4 |
| 37. | Ha avuto problemi a deglutire cibi solidi? | 1 | 2 | 3 | 4 |
| 38. | Si soffoca quando deglutisce? | 1 | 2 | 3 | 4 |
| 39. | Ha avuto problemi ai denti? | 1 | 2 | 3 | 4 |
| 40. | Ha avuto problemi ad aprire bene la bocca? | 1 | 2 | 3 | 4 |
| 41. | Ha avuto secchezza alla bocca? | 1 | 2 | 3 | 4 |
| 42. | Ha avuto la saliva appiccicosa? | 1 | 2 | 3 | 4 |
| 43. | Ha avuto problemi con il senso dell’odorato? | 1 | 2 | 3 | 4 |
| 44. | Ha avuto problemi con il senso del gusto? | 1 | 2 | 3 | 4 |
| 45. | Ha avuto tosse? | 1 | 2 | 3 | 4 |
| 46. | Ha avuto raucedine? | 1 | 2 | 3 | 4 |
| 47. | Si è sentito male? | 1 | 2 | 3 | 4 |
| 48. | E’ stato infastidito dal suo aspetto? | 1 | 2 | 3 | 4 |

Continuare alla pagina successiva

| Durante gli ultimi sette giorni: | No | Un po' | Parec-chio | Moltis-simo |
|---|---|---|---|---|
| 49. Ha avuto problemi a mangiare? | 1 | 2 | 3 | 4 |
| 50. Ha avuto problemi a mangiare di fronte alla famiglia? | 1 | 2 | 3 | 4 |
| 51. Ha avuto problemi a mangiare di fronte ad altre persone? | 1 | 2 | 3 | 4 |
| 52. Ha avuto difficoltà nell'apprezzare I pasti? | 1 | 2 | 3 | 4 |
| 53. Ha avuto difficoltà nel parlare con gli altri? | 1 | 2 | 3 | 4 |
| 54. Ha avuto difficoltà nel parlare al telefono? | 1 | 2 | 3 | 4 |
| 55. Ha avuto difficoltà ad avere relazioni sociali con la Sua famiglia? | 1 | 2 | 3 | 4 |
| 56. Ha avuto difficoltà ad avere relazioni sociali con gli amici? | 1 | 2 | 3 | 4 |
| 57. Ha avuto difficoltà ad andare tra la gente? | 1 | 2 | 3 | 4 |
| 58. Ha avuto difficoltà ad avere contatti fisici con la famiglia o gli amici? | 1 | 2 | 3 | 4 |
| 59. Ha sentito minore interesse nel sesso? | 1 | 2 | 3 | 4 |
| 60. Ha sentito minore piacere nel sesso? | 1 | 2 | 3 | 4 |

| Durante gli ultimi sette giorni: | No | Si |
|---|---|---|
| 61. Ha fatto uso di antidolorifici? | 1 | 2 |
| 62. Ha utilizzato integratori alimentari (vitamine escluse)? | 1 | 2 |
| 63. Ha fatto uso del sondino nutrizionale? | 1 | 2 |
| 64. Ha perso peso? | 1 | 2 |
| 65. Ha avuto un aumento di peso? | 1 | 2 |

© Copyright 1994 EORTC Quality of Life Study Group, version 1.0 All rights reserved
