# Supplementary material for: Sodium-hyaluronate mouthwash on radiotherapy-induced xerostomia: a randomised clinical trial
Source: Support Care Cancer. 2023 Oct 18;31(11):644. doi: 10.1007/s00520-023-08090-x (PMC10584731; doi:10.1007/s00520-023-08090-x)
Supplement: Supplementary file 6 — Supplementary file6 (DOCX 13 KB) [file 520_2023_8090_MOESM6_ESM.docx]

**Questionario di soddisfazione a seguito dell’utilizzo di Hydral ®**

*Gentile paziente, la invitiamo cortesemente a rispondere a questo questionario, il quale è stato sviluppato al fine di comprendere i maggiori pregi e le maggiori criticità a seguito dell’utilizzo dei prodotti di nostro interesse.*

*La preghiamo di indicare con una X la risposta che ritiene essere più appropriata.*

**Quanto è soddisfatto per ciascuno dei seguenti punti?**

|  | *Totalmente insoddisfatto* | *Insoddisfatto* | *Parzialmente soddisfatto* | *Soddisfatto* | *Totalmente soddisfatto* |
| --- | --- | --- | --- | --- | --- |
| **Il collutorio ha ridotto la mia sensazione di bocca secca** |  |  |  |  |  |
| **Le mucose della mia bocca mi sembrano più idratate** |  |  |  |  |  |
| **Il collutorio ha un buon sapore** |  |  |  |  |  |
| **Il collutorio dà sensazioni piacevoli dopo il suo utilizzo** |  |  |  |  |  |
| **Il collutorio è di pratico utilizzo** |  |  |  |  |  |
